# Estimation of helical angle of the left ventricle using diffusion tensor imaging with minimum acquisition time

**DOI:** 10.1186/1532-429X-16-S1-P359

**Published:** 2014-01-16

**Authors:** Ria Mazumder, Bradley D Clymer, Richard D White, Arunark Kolipaka

**Affiliations:** 1 Department of Electrical and Computer Engineering, The Ohio State University, Columbus, Ohio, USA; 2 Department of Radiology, The Ohio State University, Columbus, Ohio, USA; 3 Department of Internal Medicine - Division of Cardiology, The Ohio State University, Columbus, Ohio, USA

## Background

Myocardial fiber structure exhibits a helical geometry, characterized by the helical angle (HA). HA transitions smoothly from a negative helix in the epicardium to a positive helix in the endocardium. HA plays an important role in understanding the electrical and mechanical properties of the heart. Therefore, there exists a need to non-invasively quantify HA which can be determined using diffusion tensor imaging (DTI). The evaluation of HA measured from DTI depends on the accuracy of the diffusion tensors which in turn depend on the number of diffusion-encoding directions (DED) and the signal to noise ratio (SNR, which can be increased by increasing the total number of excitations (NEX)). However, increasing SNR and DED also increases the total acquisition time (TA). The aim of the study is to optimize the acquisition protocol (NEX and DED) to robustly estimate HA in the shortest possible TA.

## Methods

**Figure 1:**
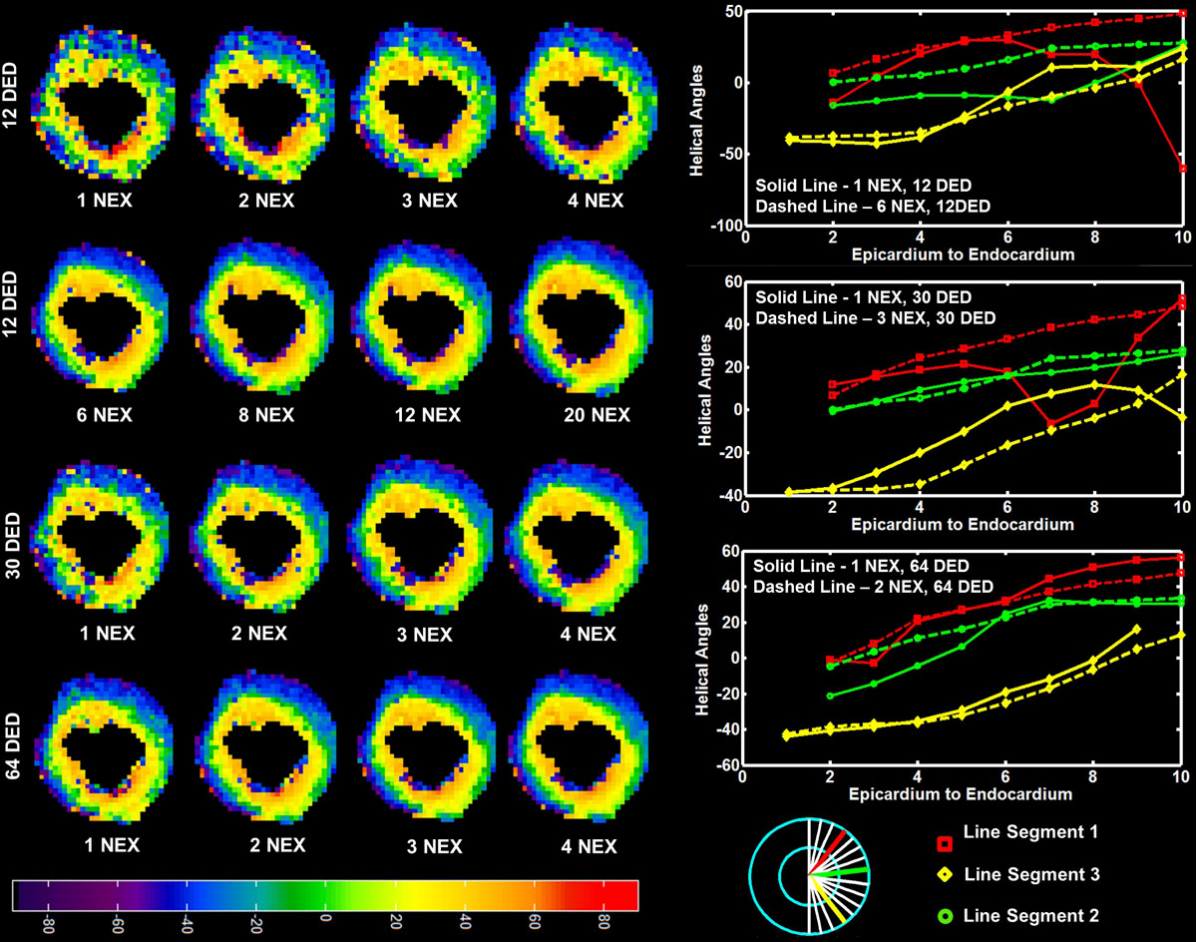
**1st, 2nd Row: 12 DED; 3rd Row: 30 DED; 4th Row: 64 DED; 1st - 4th Cols: Helical angles on a single slice extracted from the mid ventricular region of the left ventricle**. 5th Col: Line profile from three transmural line segments drawn on the free wall of the left ventricle.

Ex-vivo DTI was performed on a formalin fixed porcine heart on a 3T MRI scanner (Tim Trio, Siemens Healthcare). A diffusion-weighted spin-echo based sequence was applied to acquire multi-slice short axis view of the heart. Twenty-two repeated scans were performed with different combinations of NEX and DED detailed in Table 1. Other imaging parameters included: TE/TR = 90/7000 ms; slice thickness = 2 mm; imaging matrix = 128×128; FOV = 256×256 mm2; b-values = 0,1000 s/mm2; slices = 42. The images were masked to segment the left ventricle (LV). Custom-built software written in Matlab (Mathworks, Natick, MA) was used to obtain the diffusion tensors and estimate HA. Line profiles were computed along 16 equally spaced transmural regions (Figure 1) on the free wall of the LV to evaluate the optimum acquisition parameter required for a robust estimation of HA.

**Table 1:** Shows the acquisition time for 22 different scans, conducted with different combinations of NEX and DED.

Acquisition number	Diffusion encoding directions (DED)	Number of excitations (NEX)	Acquisition time (TA) [Mins]
1	12	1	1.63

2	12	2	3.25

3	12	3	4.88

4	12	4	6.51

5	12	6	9.76

6	12	8	13.02

7	12	12	19.52

8	12	16	26.03

9	12	20	32.54

10	30	1	3.91

11	30	2	7.81

12	30	3	11.72

13	30	4	15.62

14	30	5	19.53

15	30	6	23.43

16	30	7	27.34

17	30	8	31.24

18	64	1	8.14

19	64	2	16.27

20	64	3	24.41

21	64	4	32.54

22	256	1	32.32

## Results

Figure 1 shows HA results obtained from a mid-ventricular slice of the LV. The rows illustrate HA for a fixed DED (1st, 2nd Row - 12; 3rd Row - 30; 4th Row - 64) with varying NEX. We observed a smooth transition of the HA from the epicardium to the endocardium with increasing NEX. However, beyond a certain NEX there was no noticeable improvement in the HA transition in the transmural direction. That is, increasing the NEX beyond 6 (12 DED), 3 (30 DED) and 2 (64 DED) does not further smooth the HA transition but increases the TA. The 3 different line profiles of the same slice shown in the last column are consistent with our observation. Similar patterns were also observed in all the other slices for all the other line profiles (not shown). Furthermore, these HA are in agreement with previously published results.

## Conclusions

Our results demonstrate that a robust estimation of HA is possible using either a 12/6, or 30/3 or 64/2, DED/NEX acquisition. However, TA required for each are 9.76, 11.72 and 16.27 minutes respectively. Therefore, to obtain a robust estimate of HA in the shortest possible TA we can use a 12/6 DED/NEX combination.

## Funding

PI's start-up funds.

